# Evaluation of the Largest Series of Ultramini Percutaneous Nephrolithotomy in Preschool Children: 10-Year Experience with 711 Units of Kidney Stones

**DOI:** 10.3390/jcm14103355

**Published:** 2025-05-12

**Authors:** Mehmet Mazhar Utangaç, Onur Dede

**Affiliations:** Department of Urology, Faculty of Medicine, Dicle University, 21280 Diyarbakir, Turkey; dronurdede@hotmail.com

**Keywords:** kidney stones, percutaneous nephrolithotomy, preschool children renal units, urolithiasis

## Abstract

**Background and Objectives:** This study aimed to evaluate the safety, efficacy and outcomes of ultramini percutaneous nephrolithotomy (UM-PNL) in preschool-aged children with kidney stones. **Materials and Methods:** A retrospective analysis was conducted on 711 renal units of 676 paediatric patients aged 0–6 years who underwent UM-PNL between April 2014 and July 2024. The children’s demographic data, stone characteristics, operative details and postoperative outcomes were analysed. The procedure was performed using a 9.5 Fr sheath and a 7.5 Fr nephroscope, with laser lithotripsy applied. Postoperative follow-up included imaging and a clinical assessment of complications. **Results:** The mean patient age was 34.2 months (range: 5–72 months). Haematuria (36.8%) and urinary tract infections (24.5%) were the most common presenting symptoms. The mean stone size was 16.2 mm, and the stone-free rate was 89.2% after the first session, increasing to 96.4% with additional interventions. The mean operative time was 38 min. No major complications were observed; 8.4% of cases had Clavien grade 3b complications, most of which were managed conservatively. Blood transfusion was required in 2.6% of the cases. **Conclusions:** UM-PNL is a safe and effective treatment method for kidney stones in preschool-aged children, characterized by high stone-free rates and a low risk of complications. With proper patient selection and experienced surgical teams, UM-PNL can be considered a first-line option in paediatric stone management.

## 1. Introduction

Urinary stone disease represents a significant clinical issue in paediatric urology practice. The incidence of stones in children varies across geographic regions. Turkey is considered an endemic region for kidney stones [1]. Although the incidence of kidney stones in children is not clearly known, their frequency is gradually increasing [2]. Environmental factors, such as fluid intake, diet and obesity, contribute to this rise [3]. Kidney stones in the paediatric age group usually develop because of anatomical, metabolic and infectious causes. Therefore, the probability of kidney stone recurrence in paediatric patients is high [4,5]. Minimally invasive techniques play a vital role in the management of paediatric kidney stones. Regardless of the surgical procedure used, the aim is to ensure complete stone clearance, protect kidney function and prevent recurrence in the long term.

Extracorporeal shock wave lithotripsy (ESWL), flexible ureteroscopy (FURS) and PNL are used in kidney stone surgery. The need for anaesthesia during ESWL in children and for re-anaesthesia in cases of failure has relegated ESWL to a secondary option in the paediatric age group [6]. The use of ESWL is highly limited in this group because of children’s small body masses, short skin–kidney distances and the lack of standardisation in shockwave frequency and the number of pulses to be delivered. Flexible ureteroscopy performed in preschool-aged children is prone to complications, such as ureter injury, perforation, avulsion and ureteral stenosis, because of the narrow calyceal system and narrow ureter diameter in this population [7]. In recent years, studies have shown that PNL can be used safely, even among infants [8]. For the reasons presented above, we opted to use ultramini percutaneous nephrolithotomy (UM-PNL) instead of ESWL and FURS in our cases.

Recent advancements in endourological techniques and instrumentation have led to the widespread adoption of minimally invasive surgeries in the paediatric population [9]. To minimize complications in children, miniaturized instruments have been specifically developed [10]. With the introduction of paediatric nephroscopes, percutaneous nephrolithotomy (PNL) has become more common in the paediatric age group. In the last 10 years, PNL has become one of the preferred methods for the removal of kidney stones in patients of all ages, including infants and paediatric patients [3].

As there are no specific guidelines for the management of kidney stones in the paediatric age group, adult guidelines are often adapted for use in this population [11]. Pediatric kidneys are considerably more mobile than those of adults and are characterized by smaller renal collecting systems, thinner renal cortices, and shorter skin-to-kidney distances [12]. If large instruments are used in these patients, complications such as bleeding, renal pelvis injury, ureteropelvic junction stenosis, a loss of kidney function and sepsis may occur [13,14]. For these reasons, performing the PNL procedure in the paediatric age group is particularly challenging. No other studies in the literature have been conducted with as large a number of patients in the preschool age group as ours. To the best of our knowledge, research investigating the effectiveness of UM-PNL in the preschool age group is limited. Therefore, in this study, we aimed to present the efficacy and safety of using UM-PNL in preschool children on the basis of the procedures we performed at our centre.

## 2. Materials and Methods

Approval for this study was obtained from Dicle University’s Ethics Committee. Parental informed consent was given on behalf of the patients included in the study. Patients who underwent UM-PNL between April 2014 and July 2024 because of kidney stones were included in the study. In total, 264 of the 627 patients were girls, while 363 were boys. In total, 387 of the stones in 711 renal units (RUs) were located in the right kidney, while 324 were located in the left kidney. Patients with stone sizes > 2 cm were included in the study. Patients with stone sizes 1–2 cm were included in the study if they had symptomatic haematuria and a Hounsfield unit of >1000. Patients with skeletal deformities, obstructive renal pathologies, and renal location and position anomalies were excluded from the study. Demographic data such as the patient’s age, the location and size of the stone, laterality, the number of operations and fluoroscopy duration, and preoperative, perioperative and postoperative findings were obtained from the patients’ files retrospectively. Postoperative parameters, such as the patient’s length of hospital stay, stone clearance rate, haemoglobin decrease, complication rates, adjunctive methods and double J (DJ) stent placement, if fitted, were recorded. In the preoperative period, all patients were evaluated with a detailed history, physical examination, creatinine test, urine culture, urinalysis, kidney–ureter–bladder X-ray and ultrasonography. In case of a discrepancy between ultrasonography and the kidney–ureter–bladder X-ray, non-contrast computed tomography was performed. The operation was conducted while the urine was sterile.

Stone size was assessed based on the longest axis identified in preoperative radiological imaging; for cases with multiple stones, the total stone size was calculated as the sum of the longest axes of each stone. The decision to place a stent was based on the duration of the procedure, stone burden, and the extent of intraoperative bleeding or observed trauma. All patients were monitored postoperatively for potential complications.

Not all patients had a nephrostomy tube inserted. Considering a patient’s intraoperative condition, the duration of the operation, the residual stone burden and the number of accesses, the UM-PNL procedure was performed as total tubeless or tubeless. The nephrostomy tube was removed one day after the procedure if the drainage was clear and the patient exhibited no pain, fever, or urine leakage around the tube. Double J stents were removed under sedation anaesthesia four weeks postoperatively, while urethral and ureteral catheters were removed on the first postoperative day. Patients were discharged if there was no leakage from the PNL site following nephrostomy tube removal and if the urine was clear. Postoperative complications were assessed using the Clavien–Dindo classification.

### Percutaneous Nephrolithotomy Technique

All procedures were conducted under general anaesthesia, and all patients received prophylactic antibiotics. For prophylaxis, we used ceftriaxone 50 mg/kg/day 2 h before surgery. Prophylactic antibiotics were used until discharge. A 4 Fr retrograde ureteral catheter was placed in the lithotomy position. The catheter tip was positioned in the renal pelvis or the upper pole calyx. A 6–8 Fr Foley catheter was inserted into the urethra and secured to the ureteral catheter. The patient was then positioned prone, and gonadal shields were used to protect the gonads from X-ray exposure. Irrigation fluid heated to a temperature close to body temperature was used during the operation. Additionally, an external heater (Nellcor WarmTouch 220–240 V, 50/60 Hz, Fort Wayner, IN, USA) was used to protect the patient from hypothermia. Thus, hypothermia as a potential side effect was alleviated [14].

Retrograde pyelography was performed to determine which calyx to access. C-arm fluoroscopy collimator settings were adjusted for this age group. Monoplanar renal access was performed on the selected calyx using a 16-gauge intravenous cannula (angiocath) under fluoroscopic visualisation [15]. After urine was expelled, a flat-tipped hydrophilic guidewire with a diameter of 0.035 in and a length of 150 cm was advanced into the calyceal system, preferably to the ureter. The calyceal system was accessed over the sensor guide using a single-shot technique (12 Fr dilator). A 14 Fr sheath was used as an access sheath. In most cases, access was achieved through the lower pole calyx, but when secondary access was required, access was achieved through the middle calyx. A 14 Fr sheath and a 9.5 Fr paediatric cystoscope (12 cm long, Karl-Storz, Tokyo, Japan) were used for the UM-PNL. The stones were fragmented with a pneumatic lithotripter and laser (200–370 μm Ho:YAG laser Kuala Lumpur, Malaysia). They were fragmented to a size that could enter the sheath and were removed with a manual pressure pump. The interior of all calyces was then checked, and foreign body forceps were used, as necessary. Fluoroscopy X-ray was also used to look for residual stones, as necessary.

At the conclusion of the procedure, a 10 or 12 Fr Foley catheter was placed as a nephrostomy tube. Antegrade imaging was conducted to assess whether there was any extravasation from the collecting system. The nephrostomy tube was not inserted at the end of the operation (tubeless) in cases with an operation time of 1 h or less, a single access procedure, no intraoperative bleeding and no residual stone. The extracted stones were sent for analysis.

## 3. Results

The mean age of the patients was 34.2 months (5–72 months). The primary symptoms during clinical presentation included haematuria (262 of 711), anuria (17 of 711), urinary tract infection (UTI; 174 of 711), restlessness/vomiting (169 of 711) and persistent fever (106 of 711). Of the 711 RUs, 48 had complete staghorn stones, 105 had partial staghorn stones, 417 had renal pelvic stones and 141 had lower pole stones. The mean stone size (longest diameter) was 14.6 mm (range 10–32 mm). In the radiological evaluation of the stones, opaque stones were found in 396 kidney units and non-opaque stones in 315 kidney units. The demographic characteristics and mean stone sizes of the patients are summarised in Table 1.

Percutaneous nephrolithotomy access was performed from both the middle and lower poles in 35 patients (5%), while single access from the lower pole was performed in 676 patients (95%). Access to the upper calyx was not required in any of the patients. According to the results of the perioperative evaluation, a tubeless (with ureteral stent and without nephrostomy) catheter was inserted in 580 (81.5%) patients, while a nephrostomy catheter was inserted in 131 (18.4%) patients. An antegrade DJ stent was inserted into 261 RUs. The ureteral catheter was left on 319 RUs and removed at the 24th hour. A total tubeless procedure was not performed on any patient. The mean operation time was 38 min (19–116 min), the mean hospital stay was 1.3 days (1–8 days), and the mean duration of fluoroscopy was 34 s (22–96 s). The postoperative haemoglobin decrease was 0.32 g/dL (0.2–3 0.32 g/dL). The stone-free rate (SFR) was 89.2% according to the ultrasound performed at 1 month post-operation. Ninety-two patients required additional endourological interventions (Clavien 3b; 47 patients, ureteroscopy; 24 patients, recurrent percutaneous nephrolithotomy). After nephrostomy tube removal, urine drainage continued for more than 72 h in 22 patients (3%), and DJ stents were placed in these patients for drainage (Clavien 3b).

Stone analysis was available in 529 patients (74.4%). In the stone analysis, calcium oxalate stones (201 RUs, 38%), cystine stones (131 RUs, 24.8%), uric acid stones (68 RUs, 12.9%), calcium phosphate stones (17 RUs, 3.2%), magnesium ammonium phosphate stones (76 RUs, 14.4%) and mixed-type stones were identified (36 RUs, 6.8%).

Subfebrile fever (Clavien 1) that could be controlled with antipyretics developed in 29 patients in the early postoperative period. Fifty-seven patients developed UTIs (Clavien 2) one week after the operation, which required the use of antibiotics. Blood transfusion (Clavien 2) was performed in 19 patients (2.6%) with decreased haemoglobin. Arteriovenous fistula (AVF) (Clavien 3b) occurred in three patients (0.4%). Perioperative and postoperative complications secondary to the PNL procedure are shown in Table 2. There were no major complications or deaths in our series.

## 4. Discussion

Besides genetic aetiologies, dietary habits are among the most important factors contributing to changes in urinary biochemistry and leading to stone disease [16]. Identifying underlying risk factors and implementing targeted treatments can help reduce the likelihood of stone recurrence and prevent morbidity in children [5]. Although many patients present with multiple risk factors, in a small subset, no identifiable cause can be determined [17,18,19]. There is no consensus on the evaluation and treatment strategies for children and infants with urinary system stone disease. Kidney stone surgery has been developing in parallel with technological developments in the last few decades [20].

In the paediatric age group, the kidney is extremely mobile and can make intrarenal access difficult. Furthermore, inadvertent and drastic dilatations can cause perforations. Due to the thinner renal parenchyma in the paediatric age group, an Amplatz sheath positioned in the kidney can be easily dislodged. Ultramini percutaneous nephrolithotomy can be difficult to perform in children because the distinction between mucosal and parenchymal layers is not as clear as in adults. Performing PNL using adult devices in children raises serious concerns because of the potential risk of kidney damage [21]. Therefore, PNL was initially avoided in the preschool age group [22]. The literature emphasises that more experience is required when performing PNL with younger age groups [22].

Kidney stones in the paediatric age group have different manifestations from those in the adult age group. Fever and excessive crying are the primary symptoms, occasionally followed by haematuria, consistent with findings reported in the literature on paediatric kidney patients [13]. However, children more frequently present with gastrointestinal symptoms such as acute abdominal cramps, nausea, and vomiting, along with nonspecific signs like restlessness [22]. In our series, the patients presented to us with haematuria (262 patients), restlessness/vomiting (169 patients), UTI (174 patients), anuria (17 patients) and persistent fever (106 patients). Based on these findings, we recommend evaluating paediatric patients presenting with restlessness and urinary tract infection (UTI) for urolithiasis, particularly in regions where stone disease is endemic.

Calcium oxalate stones are the most common type in both infants and children [7]. Stone analysis in the study by Adanur Ş et al. revealed calcium oxalate (30%) and cystine stones (19%) as the most common types [20]. In our study, the most common stone types were calcium oxalate (201 RUs, 38%) and cystine stones (131 RUs, 24.8%), which is consistent with the literature. Patients were referred to paediatric nephrology along with the results of the stone analysis to prevent stone recurrence.

Percutaneous nephrolithotomy has emerged as the preferred procedure for older children with large or complex kidney stones [20]. Generally, higher SFRs (73–96%) have been reported in PNL series for the paediatric age group [13,23,24]. In a PNL study by Sen et al. on preschool children, the SFR rate was 85% [25], while it was 91% during primary PNL in the study by Patil et al. [26]. In the current study, the mean stone size was 14.6 mm (10–32 mm), and the SFR was 89.2%, similar to previous reports. Unlike the management of large stones in adults and older children, that in preschool children requires a high level of skill and experience. We believe that an SFR of 89.2% for single-tract UM-PNL monotherapy in preschool children is acceptable. The European Association of Urology 2024 Paediatric Urology Guidelines state that the definition of a “clinically insignificant residual fragment” is not appropriate for the paediatric age group, as most of the residual fragments considered clinically insignificant become symptomatic in the future and require intervention [20]. In our study, we defined “stone free” as the absence of residual fragments.

In the study by Patil et al., the operation time and fluoroscopy time were 36 min and 20 s, respectively [26]. In the study by Adanur et al., the average operation time was 50.7 min, and the average scopy time was 39 s [20]. The mean operation time in our study was 38 min, and the mean fluoroscopy time was 34 s, consistent with the literature. We attributed our short fluoroscopy and operation times to the single-shot technique and the intervention we performed using an angiocut. Taking the broken pieces out with a manual pressure pump reduces the number of entries and exits into the kidney, shortening the operation time. A fluoroscopy time of less than 1 min highlights that our study is one of the rare ones conducted in the paediatric age group. The short duration of fluoroscopy, in accordance with As Low As Reasonably Achievable principles, reveals the importance of our study [20].

Paediatric PNL involves a higher risk of hypothermia than adult PNL [24,27,28]. Pediatric patients are prone to hypothermia during prolonged procedures due to cold irrigation fluids and partial body exposure, which may also delay recovery from anaesthesia [29]. To prevent these complications, the operating room temperature should be adjusted to 22 °C, and irrigation fluids should be warmed to body temperature (37 °C) during both the preoperative and postoperative periods. We did not observe hypothermic complications in any of the patients in this study. We recommend the routine warming of the irrigation fluid and the use of external heaters during PNL, especially when the procedure is performed in the paediatric age group. We believe that this is related to our short average operation time.

The majority of complications consisted of pain, bleeding, urine leakage following nephrostomy tube removal, and postoperative fever. PNL is not a surgical procedure without complications [30]. In a study by Haberal et al., postoperative UTI was detected in 8.6% of the patients, who were then treated with appropriate parenteral antibiotics [30]. In our study, the rate of UTI was 8%, which is consistent with the rates established in previous studies in the literature. In the study by Haberal et al., the complication rate was 29.6% [30], while that in our series was 24.7%, which is compatible with the literature. Our complications were 3b and below, according to the Clavien–Dindo classification of surgical complications. The percentages of patients with Clavien grade 1, grade 2 and grade 3b complications were 4%, 10.6%, and 0.4%, respectively. These complications were low grade, consistent with the literature, and most were managed conservatively. A limited number of studies in the literature have examined the results of UM-PNL at preschool age, and our study is one of the largest series in the literature in this respect.

Bleeding is one of the most common and severe complications encountered during or after PNL. It is a significant factor influencing both patient mortality and stone-free rates (SFRs). Studies have shown that the blood transfusion rates in paediatric cases are influenced by factors such as the calibre of the instruments used, the stone burden, and the duration of the procedure [22]. In the study by Adanur et al., the postoperative haemoglobin decrease was 0.22 ± 0.10 [20], while in our study, it was 0.32 g/dL (0.2–3 g/dL), which is compatible with the literature. In the study by Haberal et al., the need for blood transfusion was 12.9% [30]. A review of the literature shows that the need for blood transfusion after paediatric PNL varies between 0% and 15.3% [20]. Our study is also consistent with the literature regarding the need for transfusion in 19 patients (2.6%). It has been reported that as the diameter of the access sheath used during paediatric PNL decreases, the amount of bleeding decreases [31].

In the study by Tepeler et al., the small size of the kidney and the greater compactness of the collecting system in children suggest that the smallest and least traumatic instruments possible should be used to reduce the risk of major complications, such as bleeding and renal trauma [31]. The most common cause of bleeding requiring treatment is vascular complications, such as renal pseudoaneurysm formation, which occurs in approximately 1% of cases [32]. The incidence of bleeding resulting from pseudoaneurysm and AVF after PNL is less than 1% [33]. In our series, AVF developed in three patients, who were then treated with embolisation. Compared to the risk of intraoperative bleeding in conventional PNL techniques, the risk in PNL is minimal, likely because of children’s smaller tract sizes, which reduce vascular injury [30].

Li et al. showed that total tubeless PNL was superior to standard PNL in significantly reducing the operative time, hospital stay and postoperative analgesic requirements, without increasing side effects [34]. Recently, some studies have reported that tubeless PNL in children shortens hospital stay and requires less analgesia [22]. In our study, 580 (81.5%) patients received tubeless catheters, and 131 (18.4%) patients received nephrostomy catheters. The discharge of tubeless patients 24 h after surgery significantly reduced hospital stay and operative costs.

In recent studies, the need for a double J stent because of urine leakage lasting longer than 48 h after PNL varies between 2.7% and 13% [30,35]. In our series, after nephrostomy tube removal, urine drainage continued for more than 72 h in 22 patients (3%), so DJ stents were placed in these patients for drainage. In our series, we attributed the low rate of DJ stent placement for prolonged urine leakage to the short operation time and thin parenchymal thickness. Double J stent placement is recommended in cases of urinary drainage from the nephrostomy tract for more than 48 h [35]. We preferred to wait for 72 h rather than 48 h because our study was conducted in the paediatric age group and anaesthesia was needed for DJ stent placement.

The main limitation of our study is that it is retrospective and not comparative. The use of ultrasonography for a postoperative stone-free status can also be considered a limitation because it cannot detect small postoperative residual fragments. Other limiting factors of our study are the lack of a statistical analysis of the data, the lack of a classification of complications according to stone size and localization, the absence of scoring systems used in PNL, and the lack of a multicentre study. Despite these, our study is one of the largest series in which UM-PNL was performed in the preschool age group. It has revealed the reliability of using UM-PNL in this patient population.

## 5. Conclusions

During PNL in preschool-aged patients, it is essential to use a small access sheath diameter, smaller-calibre paediatric instruments, and ensure that the procedure is performed by surgeons with adequate experience in adult cases. Special attention should also be given to protecting the developing body of preschool-aged children from hypothermia and radiation during the procedure. In experienced centres, high stone-free rates (SFR) can be achieved with minimal fluoroscopy and surgical times. UM-PNL is a viable and effective method for preschool-aged children, offering acceptable complication rates and high success rates.

## Figures and Tables

**Table 1:** Patient demographics and stone characteristics.

Patient Parameters	
N	627/711 renal unit
Patient age (m)	34.2 (5–72)
Gender F/M	264/363
Stone size (mm)	14.6 (10–32)
Laterality R/L	387/324
Location	
Lower pole	141
Pelvis	417
Partial staghorn (P + Inferior calyx)	105
Staghorn	48
Stone opacity	
Opaque	396
Radiolucent	315

**Table 2:** Intraoperative and postoperative parameters.

Patient Parameters	
Operative time (min)	38 (19–116)
Hospitalization time (days)	1.3 (1–8)
Fluoroscopic screening time (sec)	34 (22–96)
Double JS	261 Unit (36.7%)
Nephrostomy	131 Unit (18.4%)
Ureteral catheter	319 Unit (44.8%)
Postoperative haemoglobin decrease	0.32 g/dL (0.2–3)
Stone Free Rate (post-operative 1 month)	89.2%
Postop bleeding (transfusion required)	19 patient (2.6%) Clavien 2
Fever	29 patient (4%) Clavien 1
UTI	57 patient (8%) Clavien 2
AV fistula	3 patient (0.4%) Clavien 3b
Additional procedures	
URS	47 patient (6.6%)
Re-PNL	24 patient (3.3%)

## Data Availability

Requests to access the datasets should be directed to the corresponding author.
